# The Alteration and Clinical Significance of Th22/Th17/Th1 Cells in Patients with Chronic Myeloid Leukemia

**DOI:** 10.1155/2015/416123

**Published:** 2015-04-27

**Authors:** Ping Chen, Min Wang, Daqi Li, Yan Jia, Na He, Wei Li, Daoxin Ma, Chunyan Ji

**Affiliations:** ^1^Department of Hematology, Qilu Hospital, Shandong University, Jinan 250012, China; ^2^Department of Hematology, Jinan Central Hospital, Shandong University, Jinan 250013, China

## Abstract

T helper- (Th-) cell immunodeficiency plays important roles in tumor development and their effects in chronic myeloid leukemia (CML) remain unclear. In the present study, we mainly investigated the role of Th22, Th17, and Th1 cell and their related cytokines (IL-22, IL-17, and IFN-r) in the pathophysiology of CML. Bone marrow (BM) and peripheral blood (PB) were extracted from newly diagnosed (ND), chronic phase- (CP-) CML patients, and controls. Th subsets were examined by flow cytometry. Plasma IL-22, IL-17, and IFN-r concentrations were measured by ELISA. AHR and RORC mRNA expressions were examined by RT-PCR. The frequencies of Th22, Th17, and Th1 cells, along with the expression of specific transcription factors RORC and AHR, were significantly decreased in ND patients compared with healthy controls, while all these abnormality recovered in CP patients. In addition, there existed a significantly positive relationship between Th22 and Th17 cells in PB or BM. A significantly negative relationship was found between Th cells (Th22, Th17, or Th1) and BCR-ABL (%) IS or the number of PB white blood cells. All these results demonstrated that Th22, Th17, and Th1 cells might be important therapeutic targets in CML and could facilitate a better outcome for tumor immunotherapy.

## 1. Introduction

Chronic myeloid leukemia (CML) is a malignant hematopoietic stem cell disease characterized by the presence of Philadelphia chromosome, t(9:22)(q34:q11), resulting in the BCR-ABL fusion gene which encodes for a constitutively activated tyrosine kinase [1]. BCR-ABL gene is considered as the molecular basis of the pathogenesis of CML and as an effective indicator of diagnosis and prognosis [2]. Although the disease can be readily controlled by the introduction of ABL tyrosine kinase inhibitors (TKI), approximately one-third of patients show no response to this treatment, which seems to be associated with ABL mutation related drug resistance and the blast crisis [3, 4]. It is widely accepted that both genetic and immune factors play significant roles in the development of CML. Immune status in CML is very complex and ill-defined, and the roles of immune factors in CML have received increasing attention in recent years [5]. However, little is known about the immunopathological events, especially the abnormal T helper (Th) subsets, in the pathophysiology of CML.

Th cells play critical roles in the development and progression of inflammatory and autoimmune diseases and tumors. Th17 cells and Th22 cells are two newly described Th subsets, which have important roles in peripheral immune responses. Th17 is a unique CD4^+^ Th subset characterized by production of interleukin-17 (IL-17). Th17 cells may have evolved for host protection against microbes that Th1 or Th2 immunity are not well suited for, such as extracellular bacteria and some fungi. IL-17 is a highly inflammatory cytokine with robust effects on stromal cells in many tissues [6]. Recent data in humans and mice suggest that Th17 cells play an important role in the pathogenesis of a diverse group of immune-mediated diseases as well as in tumor. However, the role of Th17 in cancer is still being intensively discussed, with conflicting reports related to the protumoral versus antitumoral effects of these cells [7]. Recently, a novel subset of CD4^+^ Th cells, IL-22 producing T helper cells (Th22 cells), has been identified and showed to challenge the classical Th1/Th2 paradigm [8, 9]. Th22 cells are inflammatory CD4^+^ T cells that secrete IL-22 but do not express IL-17 or interferon-gamma (IFN-*γ*). Th22 cells have been shown to be important in the pathogenesis of many autoimmunity diseases. The discovery of Th17 and Th22 cells has opened up a new avenue for research into the etiology and treatment of a broad spectrum of diseases.

Recent studies have implicated the roles of Th17 and Th22 cells and their effector cytokines in the pathogenesis of several autoimmune diseases and solid tumors in humans, such as Crohn's disease, gastric cancer, and lung cancer [10]. Recent studies also reported that Th17 and Th22 cells may play vital roles in bone related diseases. Zhang et al. showed that the frequencies of both Th22 cells and Th17 cells were elevated in PB from patients with ankylosing spondylitis (AS) and rheumatoid arthritis (RA). These findings suggest that Th22 cells and Th17 cells may be implicated in the pathogenesis of AS and RA [11]. In another study, Benham et al. indicated that elevated frequencies of IL-17 and IL-22 producing CD4^+^ T cells were a feature of psoriatic arthritis [12]. CML is a kind of myeloproliferative clonal disorder of stem cell; extramedullary disease during chronic phase and blast crisis has been documented. CML in chronic phase can very rarely cause destructive bone lesions both in adults and in children and osteolytic lesions in chronic phase of CML is usually considered an impending blast crisis [13].

However, little is known regarding the roles of Th17 and Th22 cells in hematological malignancy. To date, no previous study has reported data about the immunobiology of Th subsets especially Th22, Th17, and Th1 cells in CML.

In this study, we measured the frequencies of Th cells (Th1, Th17, and Th22), the expression of their transcription factors (RORC and AHR) and their related cytokines (IFN-*γ*, IL-17, and IL-22) in PB and BM of CML patients. We also evaluated their correlations with clinical pathological characteristics.

## 2. Materials and Methods

### 2.1. Subjects and Ethics Statement

A total of 63 CML patients (26 females and 37 males; age range, 20–85 years; mean age, 47.16 ± 14.25 years) were enrolled in this study. CML patients were diagnosed according to the World Health Organization (WHO) classification [14]. Thirty-one of them were ND CML patients (13 females and 18 males; age range, 20–85 years; mean age 48.25 ± 14.99 years), and thirty-two were CP-CML patients (13 females and 19 males; age range, 22–73 years; median age 46.22 ± 13.73 years). Twenty-two age-matched healthy PB individuals (9 females and 13 males; age range 19–52 years; median age, 37.13 ± 10.22 years) were included in the study. Eleven hematologically normal age-matched BM transplant donors (4 females, 7 males; age range 21–58 years; median age, 36.63 ± 10.94 years) were used as BM controls. All cases of CP-CML were treated with imatinib mesylate. The demographic and key clinical features of CML patients are listed in Table 1. Patients with diabetes, hypertension, cardiovascular diseases, pregnant, active or chronic infection, or connective tissue diseases were excluded. Enrollment occurred between October 2013 and September 2014 in the Department of Hematology of Qilu Hospital, Shandong University (Jinan, China). Our research was approved by the Medical Ethical Committee of Qilu Hospital, Shandong University. Informed consent was obtained from all patients before enrollment in the study in accordance with the Declaration of Helsinki.

### 2.2. Flow Cytometric Analysis of Th22, Th17, and Th1 Cells

Intracellular cytokines were studied by flow cytometry to reflex the cytokine-producing cells. Briefly, heparinized whole blood (100 *μ*l) with an equal volume of Roswell Park Memorial Institute- (RPMI-) 1640 medium was incubated for 4 h at 37°C in 5% CO_2_ in the presence of 2.5 ng/mL of phorbol myristate acetate (PMA), 1 mg/mL of ionomycin, and 1.7 mg/mL of monensin (all from Alexis Biochemicals, San Diego, CA, USA). PMA and ionomycin are pharmacologic T-cell activating agents that mimic signals generated by the T-cell receptor (TCR) complex and have the advantage of stimulating T cells of any antigen specificity. Monensin was used to block the intracellular transport mechanisms, thereby leading to an accumulation of cytokines in the cells. After incubation, the cells were stained with PE-CY5 conjugated anti-human CD4 monoclonal antibody at room temperature in the dark for 20 min. The cells were next stained with FITC-conjugated anti-interferon- (IFN-) *γ* monoclonal antibody, Alexa Fluor 647 conjugated anti-IL-17A monoclonal antibody, and PE-conjugated anti-IL22 monoclonal antibody after fixation and permeabilization. All the antibodies were purchased from eBioscience, San Diego, CA, USA. Isotype controls were given to enable correct compensation and confirm antibody specificity. Fix-Perm reagents were from Invitrogen (Carlsbad, CA, USA). All samples were assayed using BD FACS Calibur Flow Cytometer. Data were analyzed with FlowJo 7.6.2.

### 2.3. Real-Time Quantitative Reverse Transcription-Polymerase Chain Reaction (RT-PCR)

The total RNA was extracted with Trizol (Invitrogen, Carlsbad, CA, USA) according to the manufacturer's instructions. Approximately, 1 *μ*g of total RNA from each sample was used to synthesize cDNA with Prime Script RT reagent Kit (Takara Bio Inc., Dalian, China). Reverse transcription reaction was done at 37°C for 15 min, followed by 85°C for 5 s. Real-time quantitative PCR was conducted using an ABI Prism 7500 Real-time PCR system (Applied Biosystems, Foster City, CA, USA) in accordance with the manufacturer's instructions. The real-time PCR contained, in a final volume of 10 *μ*L, 5 *μ*L of 2x SYBR Green Real-time PCR Master Mix, 1 *μ*L of cDNA, 3.2 *μ*L of ddH_2_O, and 0.4 *μ*L of the forward and reverse primers. The primers were shown as follows: RORC Forward 5′-CAA TGG AAG TGG TGC TGG TTA G-3′, Reverse 5′-GGG AGT GGG AGA AGT CAA AGA T-3′; AHR Forward 5′-CAA ATC CTT CCA AGC GGC ATA-3′, Reverse 5′-CGC TGA GCC TAA GAA CTG AAA G-3′. All experiments were conducted in triplicate. The PCR products were analyzed by melt curve analysis and agarose gel electrophoresis to determine product size and to confirm that no byproducts were formed. The results were expressed relative to the number of GAPDH transcripts used as an internal control. GAPDH was analyzed using the following primers: Forward 5′-GCT CTC TGC TCC TCC TGT TC-3′ and Reverse 5′-GTT GAC TCC GAC CTT CAC CT-3′. Relative gene expression level (the amount of target, normalized to endogenous control gene) was calculated using the comparative Ct method formula 2^−ΔCt^.

### 2.4. IFN-*γ*, IL-17A, and IL-22 Enzyme-Linked Immunosorbent Assay

PB and BM were collected into heparin-anticoagulant vacutainer tubes. Plasma was obtained by centrifugation and stored at −80°C for determination of cytokines. The levels of IFN-*γ* (Cat: BMS228), IL-17A (Cat: BMS2017), and IL-22 (Cat: BMS2047) were measured by enzyme-linked immunosorbent assay (ELISA) following the manufacturer's instructions (eBioscience, San Diego, CA). The lower detection limits were as follows: IFN-*γ*, 0.99 pg/mL; IL-17, 0.5 pg/mL; IL-22, 5 pg/mL.

### 2.5. Statistical Analysis

Results were expressed as mean ± SD or median (range). Statistical significance of Th subset cells, plasma cytokines, and transcription factors was determined by ANOVA, and difference between two groups was determined by Newman–Keuls multiple comparison test (*q* test) unless the data were not normally distributed, in which case Kruskal-Wallis test (*H* test) and Nemenyi test were used. The Pearson or Spearman correlation test was used for correlation analysis depending on data distribution. All tests were performed by SPSS 13.0 system. *P* value less than 0.05 was considered statistically significant.

## 3. Results

### 3.1. Th22 Cells Were Decreased in ND CML Patients and Recovered after the Treatment with TKI

**Figure 1:**
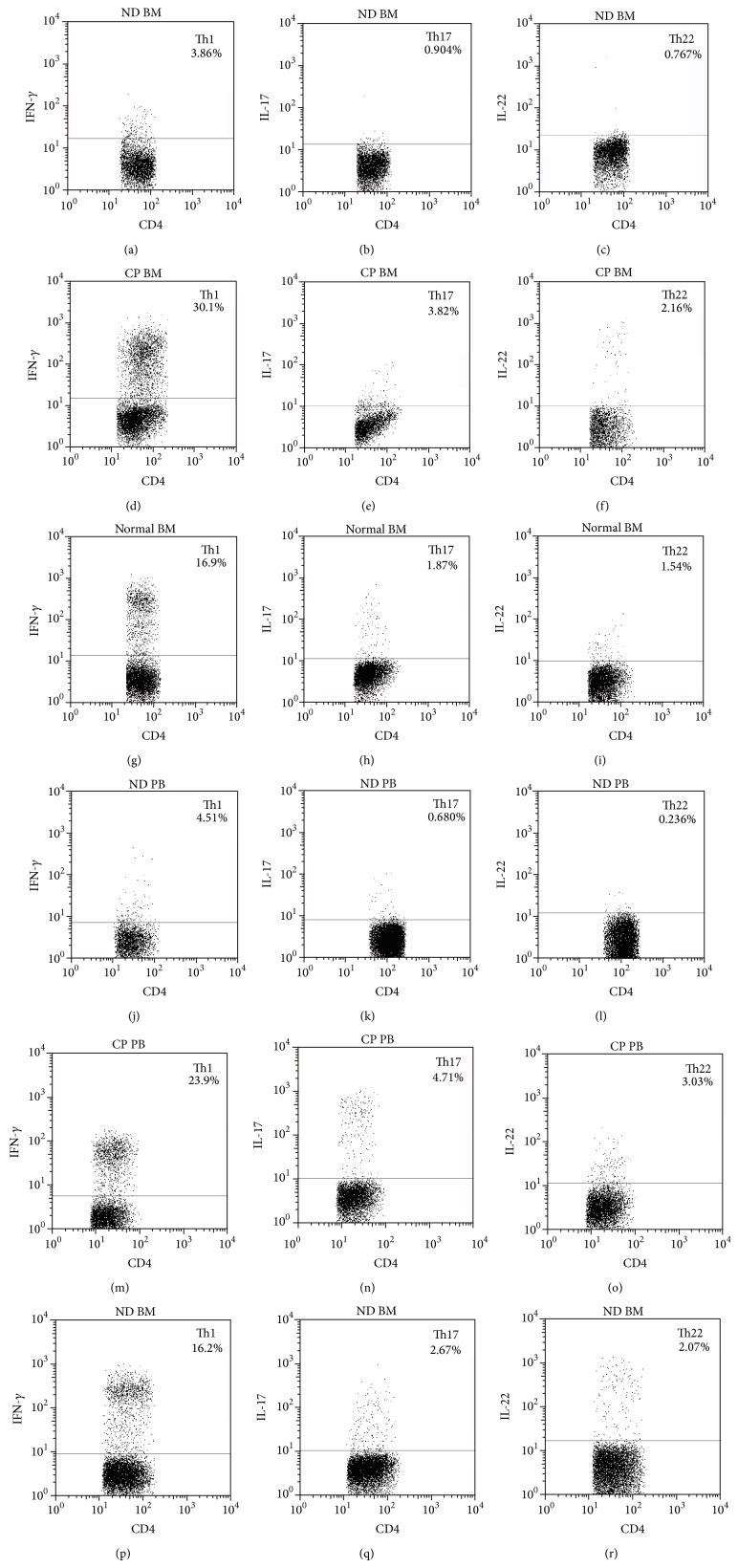
PB and BM percentages of Th1, Th17 and Th22 cells in representative ND patients, CP-CML patients, and healthy controls. (a, b, c) Representative FACS dot plots of Th1 (CD4^+^ IFN-r^+^), Th17 (CD4^+^ IL-17^+^), and Th22 (CD4^+^ IL-22^+^, IL-17^−^, and IFN*γ* ^−^) cells in BM of ND CML patients. (d, e, f) Representative FACS dot plots of Th1, Th17, and Th22 cells in BM of CP-CML patients. (g, h, i,) Representative FACS dot plots of Th1, Th17, and Th22 cells in BM of healthy controls. (j, k, l) Representative FACS dot plots of Th1, Th17, and Th22 cells in PB of ND CML patients. (m, n, o) Representative FACS dot plots of Th1, Th17, and Th22 cells in PB of CP-CML patients. (p, q, r) Representative FACS dot plots of Th1, Th17, and Th22 cells in PB of healthy controls.

**Figure 2:**
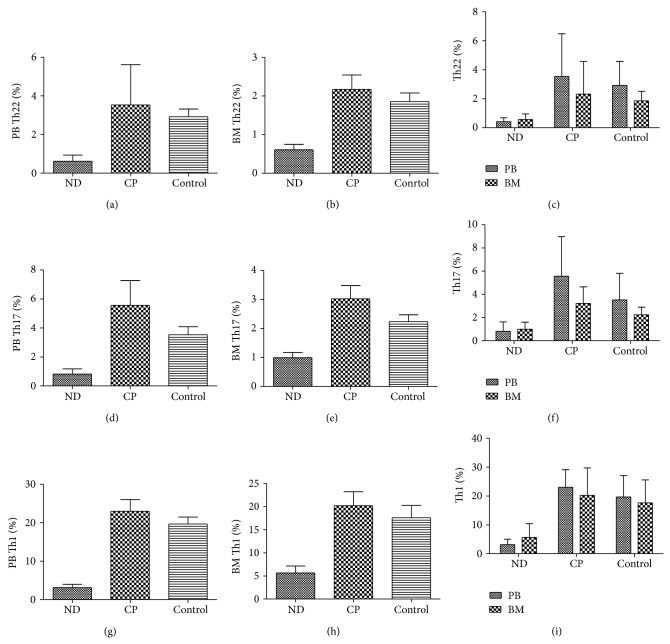
Percentages of Th17, Th22, and Th1 cells in CML patients and healthy controls. (a) Percentages of PB Th22 cells from CML patients and healthy controls. Significantly decreased percentages of Th22 cells were found in ND patients compared to CP patients (^*^ *P* = 0.029) or healthy controls (^*^ *P* = 0.005). (b) Percentages of BM Th22 cells from CML patients and healthy controls. Significantly decreased percentages of Th22 cells were found in ND patients compared to CP patients (^*^ *P* = 0.009) or healthy controls (^*^ *P* = 0.037). (c) Percentages of Th22 cells in PB or BM of ND patients, CP patients, and healthy controls. No significant differences were found between PB and BM. (d) Percentages of Th17 cells from CML patients and healthy controls. The circulating percentages of Th17 cells were profoundly decreased in ND patients compared to CP patients (^*^ *P* = 0.005) or healthy controls (^*^ *P* = 0.029). (e) The percentages of Th17 cells in BM of ND patients was proudly decreased compared to CP patients (^*^ *P* = 0.000) or healthy controls (^*^ *P* = 0.008). (f) There were no differences in the percentages of Th22 cells between PB or BM in ND patients, CP patients and healthy controls. (g) Percentages of Th1 cells from CML patients and healthy controls. The percentages of Th1 cells in PB of ND patients were proudly decreased compared to CP patients (^*^ *P* = 0.001) or healthy controls (^*^ *P* = 0.001). (h) The frequencies of Th1 cells in BM of ND patients, CP patients, and healthy controls. The frequency of Th1 cells in BM of ND patients was also significantly decreased compared to CP patients (^*^ *P* = 0.01) or healthy controls (^*^ *P* = 0.02). (i) The percentages of Th1 cells in PB or BM were not statistically different in ND patients, CP patients, and healthy controls.

**Figure 3:**
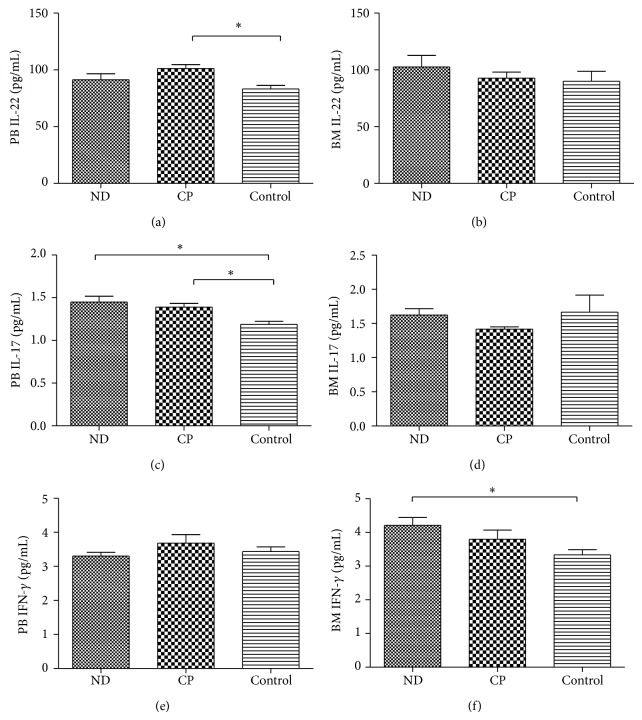
Concentrations of IL-22, IL-17, and IFN-*γ* in PB or BM plasma of CML patients and healthy controls. (a) Concentrations of IL-22 in PB plasma from CP (^*^ *P* = 0.001) patients were increased compared to in controls. No significant difference was found between ND and CP patients (*P* = 0.110) or controls (*P* = 0.194). (b) Concentrations of IL-22 in BM plasma from ND patients, CP patients, and healthy controls. No significant difference was found between ND (*P* = 0.575) or CP (*P* = 0.803) patients and healthy controls. (c) Concentrations of IL-17 in PB plasma from ND patients, CP patients, and healthy controls. The concentrations of PB plasma IL-17 in ND (^*^ *P* = 0.02) or CP (^*^ *P* = 0.01) patients were significantly higher than in healthy controls. There was no significant difference between ND and CP patients (*P* = 0.437). (d) Concentrations of IL-17 in BM plasma from ND patients, CP patients, and healthy controls. There was no significant difference between ND (*P* = 0.142) or CP (*P* = 0.645) patients and healthy controls. (e) Concentrations of IFN-*γ* in PB plasma from ND patients, CP patients, and healthy controls. No significant difference was found between ND (*P* = 0.711) or CP (*P* = 0.380) patients and healthy controls. (f) Concentrations of IFN-*γ* in BM plasma from ND patients, CP patients, and healthy controls. The concentration of BM plasma IFN-*γ* in ND patients (^*^ *P* = 0.015) was significantly higher than in healthy controls. There was no significant difference between ND and CP (*P* = 0.203) patients.

We analyzed the frequency of PB or BM Th22 (CD4^+^ IL-22^+^, IL-17^−^, and IFN*γ* ^−^) cells based on cytokine patterns after in vitro stimulation by PMA plus ionomycin in short-term cultures (Figure 1). As shown in Figures 2(a) and 2(b), the frequencies of PB or BM Th22 cells were significantly decreased in ND CML patients (0.42 ± 0.26% or 0.57 ± 0.38%; ^*^ *P* = 0.005 or ^*^ *P* = 0.037) compared to healthy controls (2.92 ± 1.65% or 1.85 ± 0.66%). Both PB and BM Th22 cells frequencies (3.53 ± 2.94%, 2.17 ± 1.17%; ^*^ *P* = 0.029, ^*^ *P* = 0.009, resp.) in CP-CML patients were statistically increased compared to ND CML patients. Although the PB and BM Th22 cells frequencies in CP-CML patients were higher than those in healthy controls, no statistical significance was observed. Meanwhile, we compared the Th22 cells between PB and B, and no difference was observed in ND or CP-CML patients. The levels of IL-22 in PB or BM of CML patients were measured by ELISA. PB IL-22 levels in CP (101.12 ± 18.29 pg/mL) patients were slightly higher than controls (83.14 ± 14.85 pg/mL; ^*^ *P* = 0.001) (Figure 3(a)). As for the levels of BM IL-22, there was no significant difference between each group (ND: 96.32 ± 26.44 pg/mL; CP: 92.67 ± 17.95 pg/mL, and controls: 89.99 ± 24.77 pg/mL) (Figure 3(b)). No correlation was found between Th22 frequencies and the levels of IL-22 in CML patients.

### 3.2. Th17 Cells Were Declined in ND CML Patients and Increased in CP-CML Patients

Similar to the findings of Th22 cells, the frequencies of PB or BM Th17 (CD4^+^ IL-17^+^) cells (Figure 1) were profoundly decreased (Figures 2(d) and 2(e)) in ND CML patients (0.82 ± 0.80% or 0.99 ± 0.60%; ^*^ *P* = 0.029 or ^*^ *P* = 0.008) compared with healthy controls (3.52 ± 2.28% or 2.23 ± 0.66%). A significant increase was also observed in CP patients (5.56 ± 3.40% or 3.03 ± 1.36%; ^*^ *P* = 0.005 or ^*^ *P* = 0.000) compared with in ND patients. We also compared Th17 cells between PB and BM and no statistical significance was found in CML patients (Figure 2(f)). The levels of PB IL-17 (Figure 3(c)) in ND (1.45 ± 0.21 pg/mL, *P* = 0.02) or CP (1.39 ± 0.22 pg/mL, ^*^ *P* = 0.01) patients were increased than controls (1.19 ± 0.17 pg/mL), while no significant difference was observed between ND and CP patients (*P* = 0.437). There was no significant difference between each BM group (ND: 1.56 ± 0.22 pg/m, CP: 1.42 ± 0.10 pg/mL, controls: 1.50 ± 0.61 pg/mL) (Figure 3(d)). No correlation was identified between Th17 frequency and IL-17 level.

### 3.3. Abnormal Th1 Cells in CML Patients

Compared with PB or BM Th1 (CD4^+^ IFN-*γ* ^+^) cells frequencies in healthy controls (19.68 ± 7.38% or 17.61 ± 7.96%), there was an observable decrease in ND patients (3.15 ± 1.92% or 5.66 ± 4.72%; ^*^ *P* = 0.001 or ^*^ *P* = 0.02). Similarly, both PB and BM Th1 cells frequencies in CP patients (23.00 ± 6.1284%, 20.22 ± 9.49%, ^*^ *P* = 0.001, ^*^ *P* = 0.01, resp.) were significantly increased than in ND patients. Nevertheless, there was no difference between CP patients and healthy controls (Figures 2(g) and 2(h)). A statistical increase of BM IFN-*γ* level was found in ND (4.21 ± 0.71 pg/mL) patients compared with in controls (3.34 ± 0.43 pg/mL, ^*^ *P* = 0.015). In addition, no statistical significance was found between ND and CP-CML patients (*P* = 0.203). There was no significant difference regarding PB plasma IFN-*γ* between each group (ND: 3.30 ± 0.33 pg/mL, CP: 3.68 ± 1.26 pg/mL, and controls: 3.44 ± 0.64 pg/mL) (Figures 3(e) and 3(f)). No correlation was observed between Th1 frequency and IFN-*γ* level.

### 3.4. AHR and RORC Expression Levels Were Downregulated Accordingly in CML Patients

**Figure 4:**
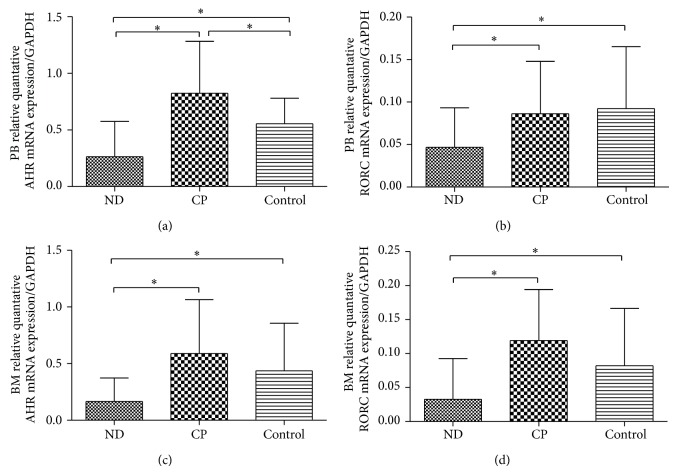
Expression of AHR and RORC mRNA in CML patients and healthy controls. (a) The expression of PB AHR mRNA was significantly decreased in ND patients compared to CP (*P* = 0.000) and healthy controls (*P* = 0.017). (b) The expression of PB RORC mRNA was significantly decreased in ND patients compared to CP (*P* = 0.048) and healthy controls (*P* = 0.034). (c) The expression of BM AHR mRNA was significantly decreased in ND patients compared to CP (*P* = 0.000) and healthy controls (*P* = 0.024). (d) The expression of BM RORC mRNA was significantly decreased in ND patients compared to CP (*P* = 0.000) and healthy controls (*P* = 0.047).

AHR, the key transcription factor directing Th22 lineage commitment, was determined by RT-PCR. Our results demonstrated that AHR was significantly decreased in PB or BM of ND CML patients (0.2647 ± 0.3103 or 0.1653 ± 0.2083) compared with CP patients (0.8243 ± 0.4582 or 0.5892 ± 0.4755; ^*^ *P* = 0.000 or ^*^ *P* = 0.000) and healthy controls (0.5560 ± 0.2245 or 0.4368 ± 0.4194; ^*^ *P* = 0.017 or ^*^ *P* = 0.024). The mRNA levels of AHR were increased in PB of CP patients compared with healthy controls (^*^ *P* = 0.012), while no statistical significance of BM AHR mRNA levels was shown between CP patients and controls (Figures 4(a) and 4(c)).

We also found that RORC transcript, the key transcription factor directing Th17 lineage commitment, was significantly decreased in PB or BM of ND CML patients (0.0466 ± 0.0464 or 0.3267 ± 0.0599) compared with healthy controls (0.0922 ± 0.0728 or 0.0819 ± 0.0842; ^*^ *P* = 0.034 or ^*^ *P* = 0.047). It was lower in ND than CP patients (0.0862 ± 0.0617 or 0.1190 ± 0.0752; ^*^ *P* = 0.048, ^*^ *P* = 0.000). No significant difference of RORC mRNA level was observed between CP patients and healthy controls (Figures 4(b) and 4(d)).

### 3.5. Th22 Cells Were Correlated with Th17 in CML Patients

**Figure 5:**
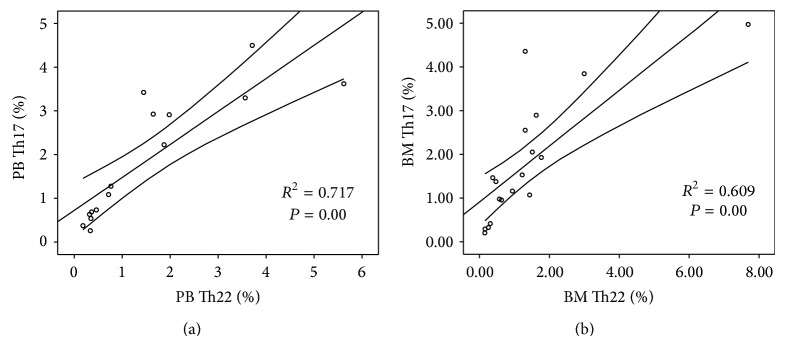
Correlation between the percentages of Th22 and Th17 cells in CML Patients. (a) Positive correlation was found between Th22 cells and Th17 cells in PB of CML patients. (b) Positive correlation was found between Th22 cells and Th17 cells in BM of CML patients.

In CML patients, a significantly positive correlation was found between Th22 cells and Th17 cells both in PB and in BM (*R* ^2^ = 0.717, ^*^ *P* = 0.00; *R* ^2^ = 0.609, ^*^ *P* = 0.00, resp.). However, Th1 cells showed no significant correlations with Th22 or Th17 cells in CML patients (Figure 5).

### 3.6. Clinical Relevance of Th22, Th17, and Th1 in CML Patients

**Figure 6:**
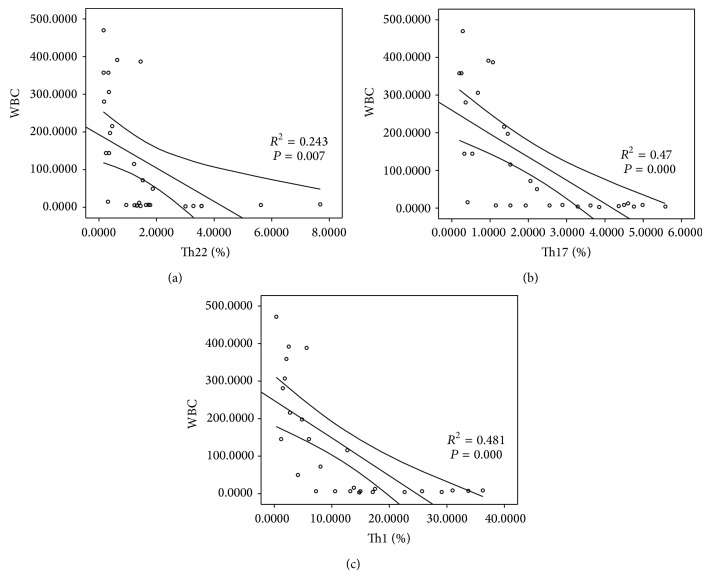
Clinical relevance between the frequencies of Th22, Th17, and Th1 cells and peripheral white blood cell number in CML patients. (a) Negative correlation was found in Th22 cell and peripheral white blood cell number. (b) Negative correlation was found in Th17 cell and peripheral white blood cell number. (c) Negative correlation was found in Th1 cell and peripheral white blood cell number.

**Figure 7:**
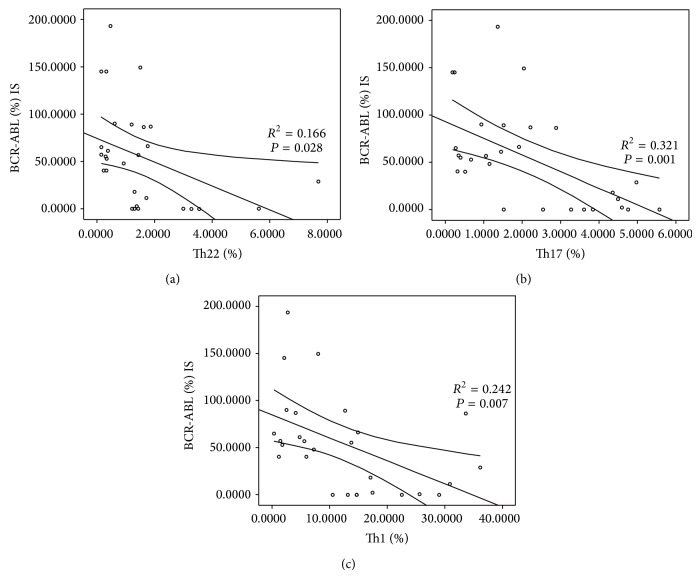
Clinical relevance between the frequencies of Th22, Th17, and Th1 cells and BCR-ABL (%) IS in CML patients. (a) Negative correlation was found in Th22 cell and BCR-ABL (%) IS. (b) Negative correlation was found in Th17 cell and BCR-ABL (%) IS. (c) Negative correlation was found in Th1 cell and BCR-ABL (%) IS.

We analyzed the association between Th cells and peripheral white blood cell counts in CML patients. The results showed that there were significantly negative correlations between Th22, Th17, or Th1 cells and peripheral white blood cell counts (*R* ^2^ = 0.243, ^*^ *P* = 0.007; *R* ^2^ = 0.47, ^*^ *P* = 0.000; *R* ^2^ = 0.481, ^*^ *P* = 0.000, resp.) (Figure 6). In addition, we also found statistical negative correlations between Th22, Th17, or Th1 cells and BCR-ABL (%) IS (*R* ^2^ = 0.166, ^*^ *P* = 0.028; *R* ^2^ = 0.321, ^*^ *P* = 0.001; *R* ^2^ = 0.242, ^*^ *P* = 0.007, resp.) (Figure 7).

## 4. Discussion

CML represents 15% of newly diagnosed leukemia cases in adults in China [5]. Recent studies have shown that T-cell immunodeficiency is a common feature in cancer patients, which may relate to initiation and development of tumor [5] and immunotherapy may become a kind of effective method to control tumor growth and recurrence. Therefore, understanding the immune status especially the Th-cell immune function in patients with CML is very important and essential to make effective immunotherapy. However, to date, there are no sufficient data regarding the specific roles of Th cells in the development and progression of CML.

Th22 cells are distinct from other Th cells, such as Th1, Th2, and Th17, indicating that Th22 have an individual signature. IL-22, as the main effector cytokine of Th22, belongs to the IL-10 cytokine family [15]. Recent studies have implicated the role of Th22 and IL-22 in the pathogenesis of a variety of solid tumors and a limited number of hematological malignancies including AML [16], MDS [17] and ALL [18]. No data has been reported regarding the role of Th22 cells and IL-22 in the pathogenesis of CML. Therefore, the present study aimed to determine the levels of Th22 cells and IL-22 and its specific transcription factor AHR in CML patients. Our results demonstrated that the percentages of PB and BM Th22 subset were significantly decreased in ND CML patients compared to healthy controls, while Th22 cells may attain a certain degree of recovery when the patients achieved complete remission. Moreover, we further investigated the expression of AHR, the key transcription factor directing Th22 lineage commitment, and found that AHR mRNA expression also significantly decreased in PB and BM of ND CML patients. All these results suggested that downregulation of Th22 cellular immunity and impaired Th22 immune function may contribute to the occurrence and development of CML.

Considering the involvement of IL-22 in the regulation of cell growth, proliferation, and cell cycle control, it is conceivable that IL-22 might play an acceleration or inhibition role during tumorigenesis [19]. However, in our study, the levels of IL-22 have been found comparable in each group of CML patients. And there is no connection between Th22 and IL-22. As we know that, in humans, adaptive immune cells of the immune system, such as Th22 and Th17, are the major T-cell subsets producing IL-22 [15, 20, 21]. IL-22 is also expressed by CD8^+^ T cells [22, 23] and other innate lymphocytes, including NK22 subset of natural killer cells [24, 25], and CD11c^+^cells [23, 26]. Our results may suggest that, in CML, Th22 cells may only account for a minor proportion in producing IL-22 and probably other kinds of cells made up this difference.

Although the role of Th17 in autoimmune diseases and infection has been relatively well documented, the impact of Th17 in cancer remains difficult to ascertain [7]. The current data regarding the role of Th17 cells in pathogenesis of cancer are scarce and controversial [27]. There are several researches about the roles of Th17 in hematological malignancies including multiple myeloma [28] and CLL [29], AML [16, 30], and non-Hodgkin lymphoma [31]. It has been reported that serum levels of IL-17 are increased in multiple myeloma patients and are correlated with disease prognosis, which suggested IL-17 as tumor-promoting factor in multiple myeloma. Th17 was found to be accumulated in the bone marrow of multiple myeloma [28]. Another study indicated that progression of CLL is associated with downregulation of IL-17-producing T cells, implying contribution of these subsets of T cells in the progression of CLL [29].

In the present study, similarly the results of Th22 cells, we showed that the frequency of Th17 was significantly decreased in ND CML patients compared to healthy controls. The RORC expression was also dramatically reduced in ND patients. Moreover, both Th17 frequency and RORC expression all recovered to the normal level in CP-CML patients. These results may suggest that Th17 cells may play a protective role in CML pathogenesis and their potential implication in immunotherapy of this malignancy.

Th1 cells have been linked to the development of autoimmune inflammatory processes. Th1 cells secrete a large number of IFN-*γ* and promote the cell-mediated immunity [32]. We also detected the frequencies of Th1 cells and related cytokine IFN-*γ* in CML patients. In accordance with the variation of Th22 and Th17 cells, the immunobiology of Th1 cells was damaged in ND CML patients and recovered after the treatment with TKI.

In addition, there existed a positive correlation between Th22 and Th17 subsets both in PB and in BM of CML patients, implying that differentiation of Th22 and Th17 cells may be induced in an influential manner in CML. This coreduced frequencies of Th22 and Th17 cells suggested that these two T-cell subsets may play a synergistic role in leading to a dramatic immunodeficiency in CML patients.

We analyzed the association between Th cells and peripheral white blood cell counts or BCR-ABL (%) IS in CML patients. Our results showed that there was significantly negative correlation between Th22, Th17, or Th1 cells and peripheral white blood cell counts or BCR-ABL (%) IS. These data may suggest that the proportions of Th22, Th17, and Th1 cells are associated with tumor burden in CML patients and may be useful in evaluating therapeutic effects.

In conclusion, we firstly demonstrated that the frequencies of Th22, Th17, or Th1 cells and their specific transcription factor expression are significantly reduced in ND CML patients both in PB and in BM. Furthermore, these cells immunodeficiency may recover after the treatment with TKI. All these results indicate that these Th subsets may be involved in the pathogenesis of CML and may become effective therapeutic targets for CML patients.

## Figures and Tables

**Table 1:** The characteristics of subjects.

	ND CML	CP-CML	Healthy control
	(*n* = 31)	(*n* = 32)	(*n* = 33)
Age (years)	48.25 ± 14.99	46.22 ± 13.73	37.00 ± 10.28
Gender (male/female)	18/13	19/13	20/13
WBC (∗10^9^/L)	189.74 ± 116.37	6.10 ± 1.69	6.37 ± 2.52
HB (g/L)	103.93 ± 24.26	134.33 ± 17.55	128.25 ± 14.35
PLT (∗10^9^/L)	505.12 ± 323.68	209.16 ± 91.25	215.25 ± 115.64
BCR-ABL (%) IS	61.16 (27.51–183.9)	0.056 (0.000–28.85)	

ND: newly diagnosed; CP-CML: chronic phase-CML; WBC: white blood cell; RBC: red blood cell; HB: hemoglobin; PLT: platelet.
